# Split G-Quadruplexes Enhance Nanopore Signals for Simultaneous Identification of Multiple Nucleic Acids

**DOI:** 10.1021/acs.nanolett.2c01764

**Published:** 2022-06-07

**Authors:** Jinbo Zhu, Filip Bošković, Ulrich F. Keyser

**Affiliations:** Cavendish Laboratory, University of Cambridge, JJ Thompson Avenue, Cambridge CB3 0HE, United Kingdom

**Keywords:** Nanopore, G-quadruplex, nucleic acid detection, multiplex sensing, DNA nanostructure

## Abstract

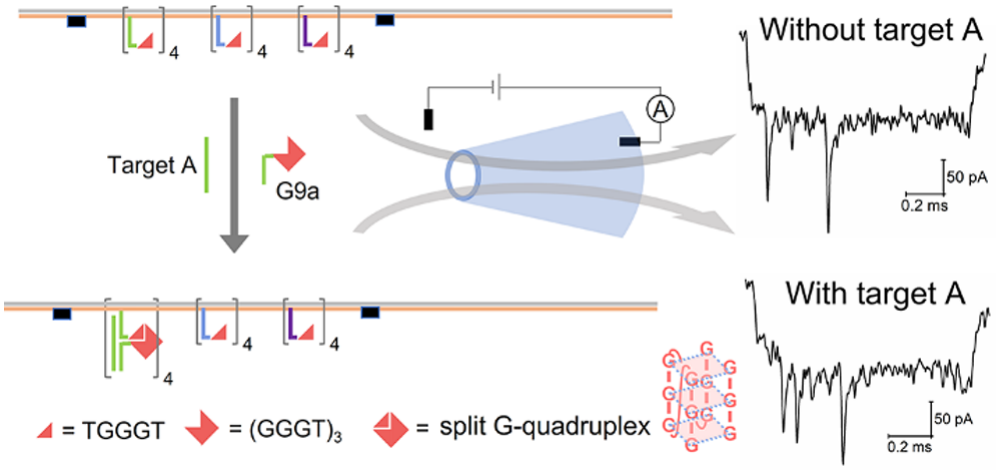

Assembly of DNA structures based on hybridization like split G-quadruplex (GQ) have great potential for the base-pair specific identification of nucleic acid targets. Herein, we combine multiple split G-quadruplex (GQ) assemblies on designed DNA nanostructures (carrier) with a solid-state nanopore sensing platform. The split GQ probes recognize various nucleic acid sequences in a parallel assay that is based on glass nanopore analysis of molecular structures. Specifically, we split a GQ into two asymmetric parts extended with sequences complementary to the target. The longer G-segment is in solution, and the shorter one is on a DNA carrier. If the target is present, the two separate GQ parts will be brought together to facilitate the split GQ formation and enhance the nanopore signal. We demonstrated detection of multiple target sequences from different viruses with low crosstalk. Given the programmability of this DNA based nanopore sensing platform, it is promising in biosensing.

## Introduction

The DNA G-quadruplex (GQ) is a unique four-stranded DNA structure that is important in biologic processes^1,2^ and also widely applied in bioanalytical science.^3,4^ There are two major ways to apply GQ for biosensing. First, the GQ can selectively recognize specific metal ions, small molecules, and proteins.^5^ GQ structure has been found in many G-rich aptamers, so GQ itself is a powerful sensing probe.^6^ Second, GQ can cooperate with specific ligands to act as a reporter in sensing techniques.^7^ One prominent example is GQ fluorescent ligands that lead to enhanced fluorescence upon binding. The presence of a target analyte will cause the formation of GQ or alteration of the conformation that is read out by the change of fluorescence signal. Such fluorescent assays have been widely used for nucleic acid detection.^8^ To improve the sensitivity for discrimination of single nucleotide polymorphisms, fluorescent and colorimetric assays based on a split GQ sensing strategy were proposed and developed over the past decade.^9−12^ However, these GQ sensing strategies have limited multiplexing ability, and suitable ligands are required.

The fluorescent or colorimetric readout can be replaced by resistive-pulse sensing with nanopores. Nanopores are a powerful sensing platform to directly investigate the formation and conformation change of GQ on a single molecule level without a specific ligand or label. Protein nanochannels were used to monitor the folding of GQ^13−15^ and can even detect ions and ligands which can stabilize the four-stranded structure.^16−18^ Si_3_N_4_ nanopores were also reported to be able to detect the formation of GQ.^19,20^ Recently, glass nanopores with ∼5 nm diameter were applied by us to monitor the folding of GQ on a long (7228 bp) double-stranded DNA carrier.^21,22^ The DNA carrier is normally constituted of an M13mp18 scaffold and 190 short oligonucleotides.^23,24^ This self-assembled nanostructure enables multiplexing by either designing specific binding sites or adding structures for digital encoding.^25−27^ However, the glass nanopores with diameter smaller than 10 nm are difficult to fabricate, and lifetimes can be limited, which is unfavorable for fast detection and wide usage.

Nucleic acid tests are of great interest for clinic molecular diagnosis highlighted by the current global spread of novel coronavirus (SARS-CoV-2).^28^ The split GQ-based detection method is label-free, sensitive, and easy to adjust to different targets, and split GQ could be used for enhancing the nanopore signal.^29^ Herein, we combine a split GQ assay, glass nanopores (∼14 nm), and DNA carriers to achieve multiplexed nucleic acids identification with single-base resolution. Three short DNA sequences from different types of viruses, SARS-CoV-2 and influenza A virus subtypes H1N1 and H5N1, were tested simultaneously. The target nucleic acid strand can be accurately captured by the two GQ parts of the split GQ (sGQ) based on the multicomponent probe approach. The amplitude of the ionic current signals is composed of up to five identical split GQs that form upon target strand hybridization, which amplifies the magnitude of the current blockade even with glass nanopores with 14 nm diameter. The DNA carrier with multiple binding locations was utilized to distinguish the signals from different targets bound on the corresponding positions. Compared with our previous DNA sensing assays built on the carrier and nanopore platform,^25,30^ the split GQ method avoids protein labels and strand displacement reactions, which greatly simplifies detection.

## Results and Discussion

**Figure 1:**
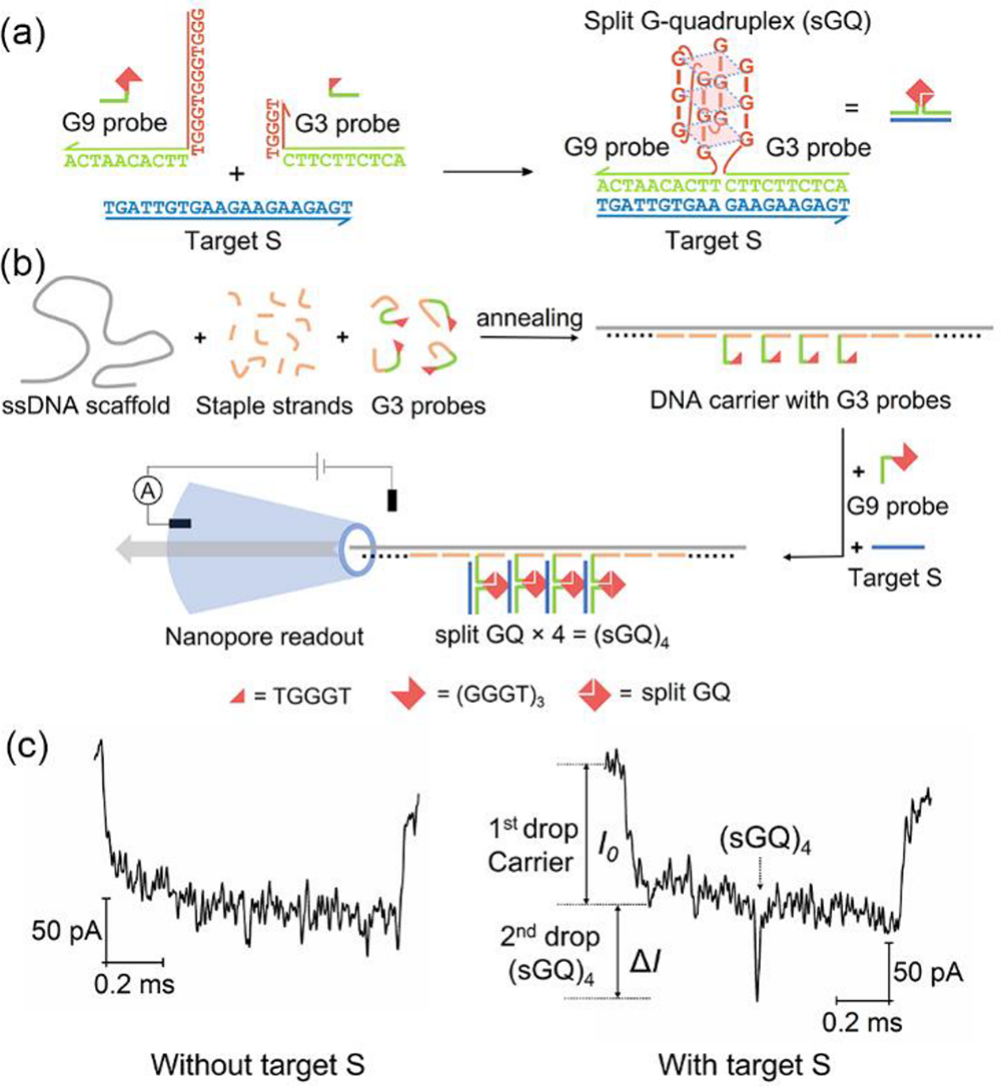
Detection of target strand S via split GQ (sGQ) reformation and readout by solid-state nanopore. (a) Schematic of the split GQ assay for nucleic acid detection of target S. sGQ assembly is facilitated by binding of G3 and G9 probe to S. (b) Schematic of assembly of DNA carrier with G3 probes and translocation of a carrier with four adjacent (no other staples between them) split GQs [(sGQ)_4_] in the middle of the DNA carrier. DNA carrier structure is read out by resistive-pulse sensing with a glass nanopore. (c) Typical nanopore current signals of the DNA carrier with four G3 probes in the absence (left) or presence (right) of the target strand S. The concentrations of DNA carrier, strand S, and G9 were 0.25 nM, 20 nM, and 24 nM, respectively, in the nanopore measurement. Nanopore measurement was performed in Tris-LiCl buffer (10 mM Tris-HCl, 4 M LiCl, 20 mM KCl, pH 9.0).

First, we explored the possibility of applying larger glass nanopores to detect split GQ on DNA carriers. Figure 1a depicts the idea of the split GQ. An intact GQ with 12 guanine residues is split into two 3:9 parts asymmetrically and distributed onto the two probe strands G3 and G9,^10,31^ which are partially complementary to the target strand S (Table S1, S2). We choose the 3:9 split mode rather than 4:8 or 6:6^10−12^ in order to minimize the background nanopore signal of short G probes on the carrier without a target, because the longer the overhang on the carrier is, the stronger the current blockage would be. In the presence of target S, G3 and G9 can hybridize with it and get close to each other to form a split GQ. The target triggered GQ formation was confirmed by the fluorescent turn-on ligand NMM (*N*-methyl mesoporphyrin IX) (Figure S1).^32^ One typical DNA carrier design with split GQ sensing components in the middle is designed and assembled as indicated in Figure 1b. A long single strand DNA (7.2 kb) is linearized by short DNA staple strands, and selected staples are extended with target capturing sequences as overhangs on the carrier for sensing. We have shown that a single GQ is too small to be detected by 14 nm glass nanopores.^21^ To facilitate sensing with 14 nm nanopores, a group of four adjacent G3 probes in the center of the carrier. The identical binding sites located in the middle of the DNA carrier allows for specific detection and simplifies data analysis. Only after addition of the longer G9 probe and target strand S, four split GQs form [abbreviated as (sGQ)_4_] as indicated in Figure 1b. DNA carriers passing through the nanopore give rise to signals as depicted in Figure 1c. The first level current drop (*I*_0_) indicates the DNA carrier and the absence or presence of an additional peak indicates the absence or presence of the target. In the absence of target strand S, G3 on the carrier and free G9 in solution cannot form a split GQ on the carrier. We use one threshold to distinguish if the second current drop (Δ*I*) is counted as positive detection (Figure 1c). In Figure 1c we show two typical events with and without target S. We observe a clear peak in the middle only when an excess of S was added (20 nM S compared to 0.25 nM carrier). Details of the target binding protocols are given in Supporting Information Section S1.4. The peak indicates the formation of split GQs upon target binding. From our experiments we chose a threshold of 0.3 for the relative peak intensity (Δ*I*/*I*_0_) to determine the number of events with (sGQ)_4_. We define the occupied fraction (OF) as the number of events with peak divided by the number of total unfolded translocation events. With a threshold of 0.3 we found a clear difference of OF before (15.5%) and after (89.6%) the addition of the target S (Table S8). This result indicates the split GQ based nanopore sensing method can detect the DNA target S. The relationship between target concentration and OF is shown in Figure S2.

**Figure 2:**
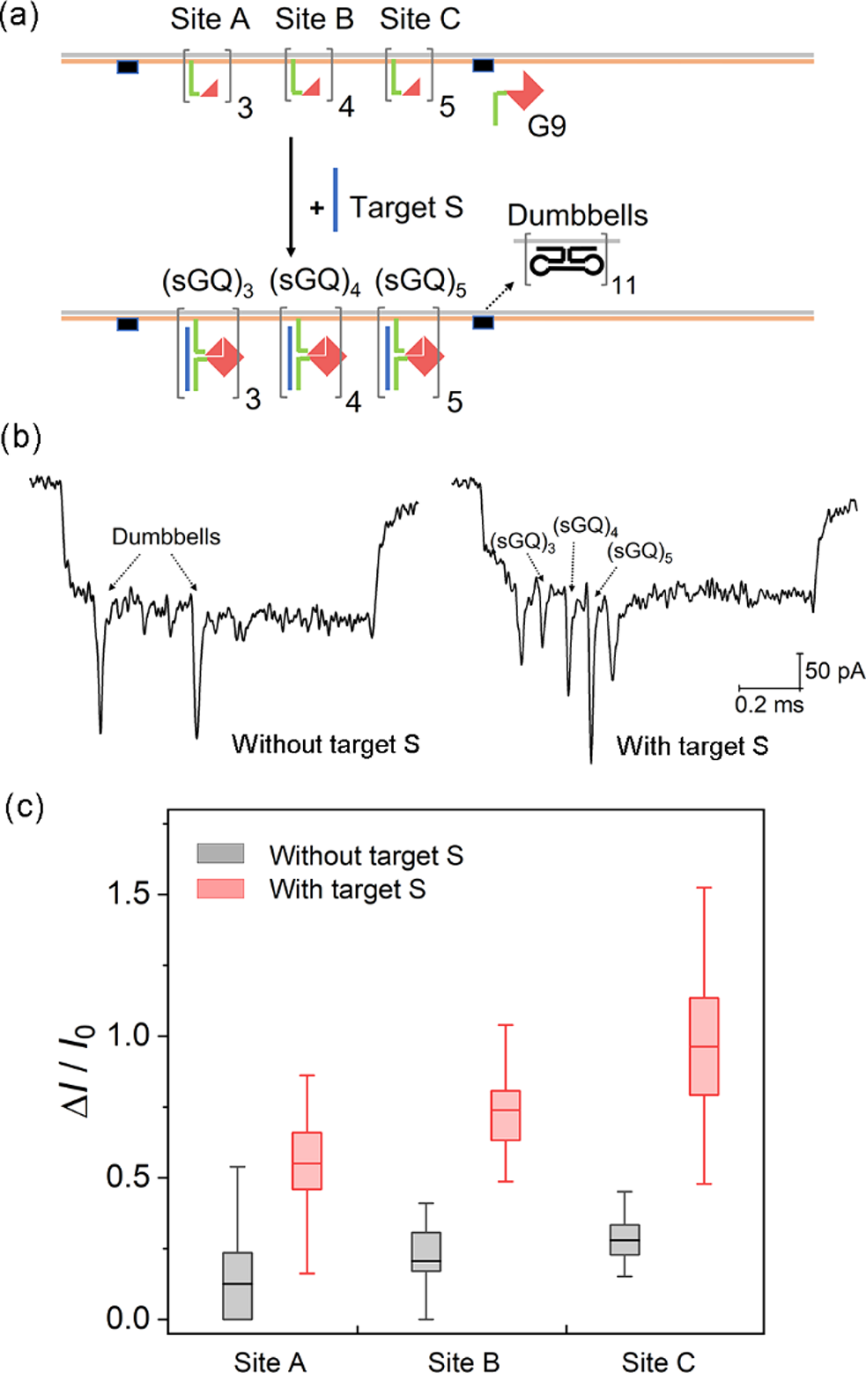
Optimization of the numbers of split GQ (*n*) on carrier for DNA target sensing. (a) Design of the carrier for optimizing the number of G3 probes at each sensing site. Two groups of DNA dumbbells are designed on the carrier for referencing of multiple binding sites. (b) Typical nanopore current signals of the carrier in the absence (left) or presence (right) of DNA target S. (c) Box chart showing the relative peak intensities (Δ*I*/*I*_0_) of different numbers of sGQ at the three sensing sites based on the analysis of 50 unfolded events from the two samples: without and with target S. Histograms of Δ*I*/*I*_0_ at the three sites are given in Figure S3. The two samples were measured by the same nanopore. The mean values are represented by a line across the boxes, and the whiskers span ±1.5 IQR (interquartile range).

For the split GQ based nanopore sensing platform, a key parameter is the number of adjacent G3 probes on the carrier. We determined the number of G3 strands that can offer the best compromise between relative peak intensity (Δ*I*/*I*_0_) with and without target. Addition of G3 strands leads to a higher background signal even in the absence of target, because the nanopore acts as a volumetric sensor and the single-stranded DNA will also block the ionic current. Hence, we designed a carrier with multiple sensing sites as shown in Figure 2a. The asymmetric design of carrier facilitates the judgment of the direction of the translocation. Three groups of G3 probes for the same target S are placed between two referenced DNA dumbbells structures (double hairpins),^23^ which are useful for locating and identifying the target signals. The distances between these binding sites are the same (Tables S3–S7), so we can identify the signal based on its position in the event (appearance time during the translocation).

Three, four, and five adjacent G3 probes are immobilized at the three sensing sites A, B, and C of the carrier, respectively (Figure 2a, top). In the absence of S, Δ*I*/*I*_0_ of most peaks observed between the two reference structures are less than 0.3. A typical event is shown in Figure 2b, left. When S was added, three peaks caused by the different numbers of split GQs [(sGQ)_3_, (sGQ)_4_, and (sGQ)_5_] appeared at the sensing area (Figure 2b, right). More sample events can be found in Figure S11. On average, the peak intensities increased with the split GQ numbers, as expected. As indicated in Figure 2c and Figure S3, a clear growth of Δ*I* is observed with the increasing G3 numbers at the three sensing sites on the carrier. When the G3 number is four, we can already separate the target strand induced signal (red) from the background (gray) by a threshold of 0.3 for Δ*I*/*I*_0_. However, for the (sGQ)_5_, although the signals can still be separated, the intensity of the blank sample increased close to the threshold, and there is overlap between the target absent and present measurements. Additionally, more G3 probes are needed for (sGQ)_5_, which will make the carrier more complicated, especially for multiplexed sensing. Thus, for our experimental conditions, (sGQ)_4_ with four adjacent G3 probes offers the best signal–noise readout to detect the target DNA with a threshold of (Δ*I*/*I*_0_) < 0.3.

The significance of the GQ structure for the creation of the nanopore signal is shown in Figure S4. We replaced the split GQ forming domain with a short double strand (10 bp) to make a DNA three-way junction (3WJ) with similar molecular weights (Table S1 and Table S4). Four adjacent 3WJ and four adjacent split GQ were placed on a same carrier for direct comparison. (sGQ)_4_ shows more enhanced peak currents than (3WJ)_4_, which indicates that the use of (sGQ)_n_ is crucial for enhancing the current signal and signal-to-noise ratio.

The effects of ions on the nanopore-based DNA sensing approach are also investigated. Since K^+^ can efficiently stabilize the GQ structure,^33,34^ we studied how K^+^ affected the DNA sensing by fluorescent turn-on ligand NMM and nanopore. As shown in Figure S5a, the K^+^ is essential for GQ formation and NMM fluorescence enhancement. Li^+^—the main cation in our nanopore buffers—contributes little to the fluorescence intensity. We obtained similar results with nanopore measurements of four split-GQs on the DNA carrier. In both bulk and single-molecule measurements, K^+^ is vital for the GQ detection (Figure S5b). Our experiments also indicate that fluorescence detection can be replaced by molecular analysis with glass nanopores for studying the interaction between GQ and different ions or ligands.

**Figure 3:**
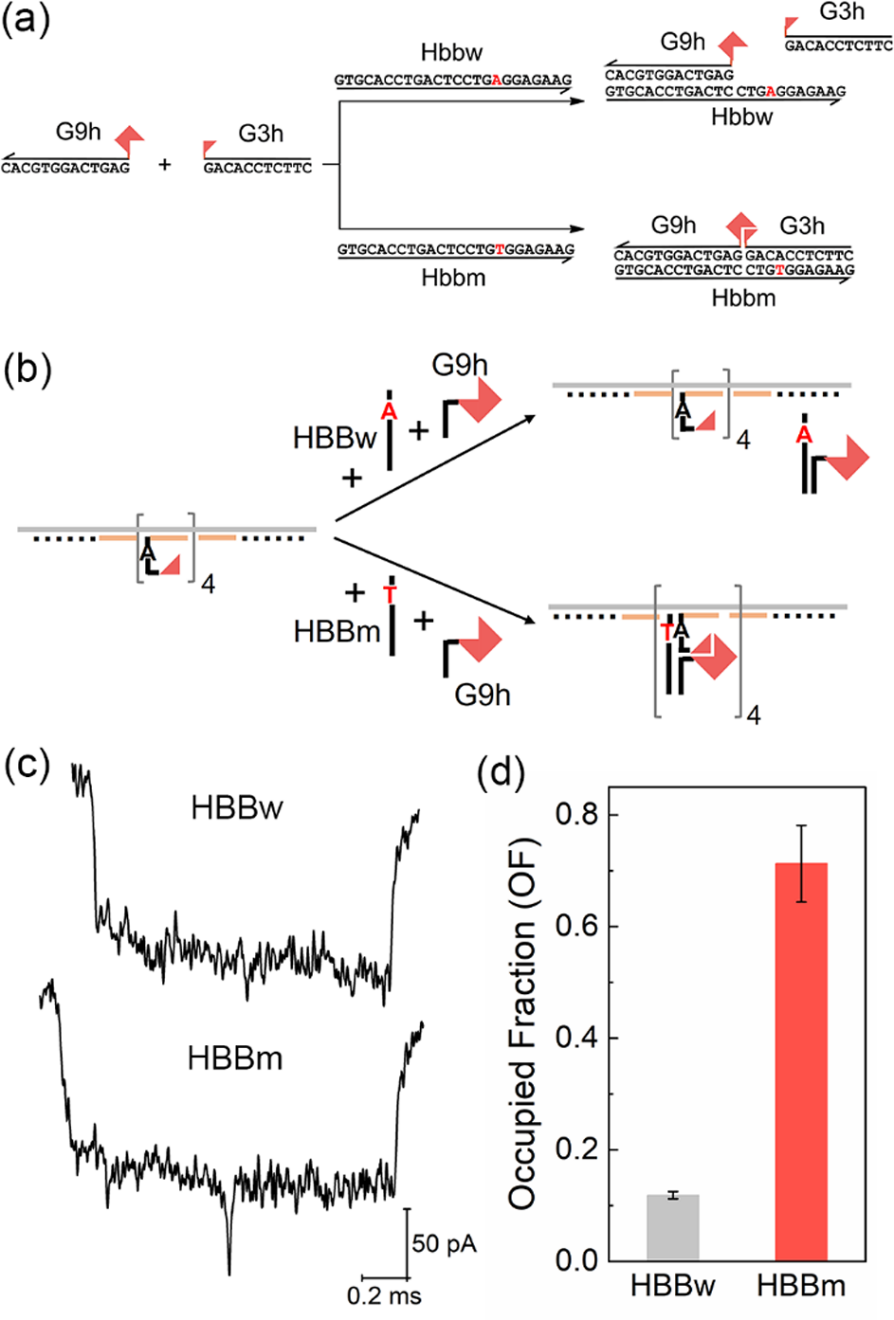
Detection of the point mutation in β-globin gene (HBB) sequence by the split GQ based nanopore sensing method. Schematic diagram is given in (a) for split GQ sensing strategy and (b) for carrier design, example events are in (c) and bar graph of the occupied fractions (OFs) for different targets are in (d). HBBw and HBBm are the wild and mutant target sequences of the HBB gene, respectively. The 20 nM HBBw or HBBm was mixed with 0.25 nM carrier and 24 nM G9h. The error bars (standard errors of mean) are obtained from three repeated nanopore measurements. Detailed nanopore data can be found in Table S8.

Furthermore, the split-GQ-nanopore sensing method enables single nucleotide mutation detection. We chose the well-known point mutation (nucleotide A > T) in β-globin gene (HBB) as an example.^35^ The split GQ-based sensing strategy for detection of single nucleotide mutation in HBB is illustrated in Figure 3a. Hybridization of target and probes was verified by native polyacrylamide gel electrophoresis as shown in Figure S6. As shown in Figure 3b, we add four G3 probes for capturing the mutated HBB segment (HBBm) in the middle of the carrier. Events with the central peak were mainly observed in the sample with HBBm (Figure 3c), as expected. More sample events can be found in Figure S12. In Figure 3d, the clear difference of the OF between wild (11.8%) and mutant (71.3%) groups of the HBB gene proves the split GQ based nanopore sensing assay can accurately identify single nucleotide mutation, which also indicates sequence specificity that is required for multiplexed detection.

**Figure 4:**
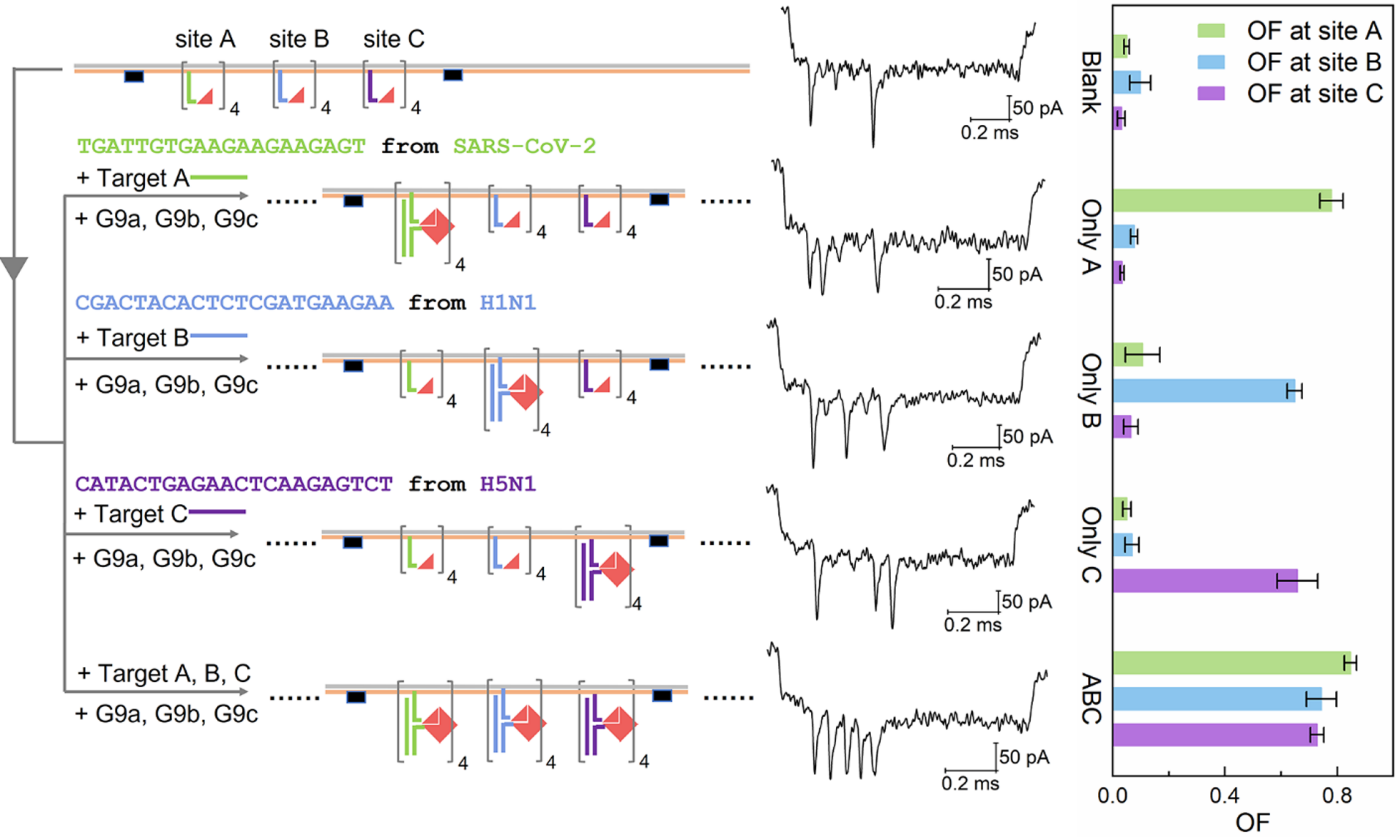
Multiplex detection of three DNA targets A, B, and C using one carrier with target-specific groups of G3 probes. Construct of the carrier is shown at the top. Sequences of A, B, and C are from SARS-CoV-2, H1N1, and H5N1 viruses, respectively. G3 probes on the three sensing sites A, B, and C on the carrier and G9 probes G9a, G9b, and G9c in the solution are designed to hybridize with the target strands A, B, and C, respectively. Sample events and OF for each situation are given on the right. The blank control without any targets is shown in the top row (Blank). Targets A, B, and C were separately detected and are shown in the second, third, and fourth rows (only A, only B, and only C), respectively. Three targets were detected simultaneously and are shown in the bottom row (ABC). The concentrations of target, carrier, and G9 probes are 20 nM, 0.25 nM, and 24 nM, respectively. The error bars (standard errors of mean) are obtained from three repeated nanopore measurements. Detailed nanopore data can be found in Table S9, and more sample events are given in Figure S13.

Based on the above analysis, we built a multiplexed sensing platform to detect three different DNA targets from viral sequences at the same time. The design of the DNA carrier is shown at the top of Figure 4. Three groups of G3 probes for the different targets (Tables S3–S5) are located at three sensing sites A, B, and C on the carrier, respectively. They are designed for capturing the target DNA strands A, B, and C (Table S1), whose sequences are from the genomes of coronavirus SARS-CoV-2 and influenza A virus subtypes H1N1 and H5N1, respectively.^36^ In the absence of targets, no obvious peaks can be observed between the two reference peaks for the sample event and bar charts in Figure 4, top row, and Table S9.

One of the three targets, strand A is first detected alone to test the sensing capability and selectivity of this platform. Strand A was premixed with its G9 probe (G9a, Table S1) and then added to incubate with the carrier for 10 min before nanopore measurement. As shown in the second row of Figure 4, when A·AG9 was present in the solution, only one obvious peak close to the first reference structure was observed in most events, and the OF at site A (77.9%) was much higher than the other two (7.6% for site B and 3.3% for site C) (Table S9). Target strands B and C were also detected separately using the same carrier, and similar results were obtained as shown in the third and fourth rows of Figure 4, respectively. The above results demonstrate that this multiplexed sensing platform can detect the target strand by counting the downward peaks at the specific sensing site, and no apparent crosstalk was observed on the other sites. A sample with all three target DNA strands was also tested in the same way, and the result is shown in the bottom row of Figure 4. As expected, the OFs of >0.7 were obtained on the three sensing sites. Thus, three different sequences from three viruses can be detected at the same time.

Another important feature of this sensing method is that we can detect several different segments from the same virus to ensure the detection accuracy. Taking SARS-CoV-2 as our example, three groups of G3 probes for target A and another two DNA targets D and E from the same genome were designed on the carrier as shown in Figure S7a. There is no signal interference when A was added individually (Figure S7b), and the three targets can be detected simultaneously (Figure S7c). The multipoint detection approach has the potential to dramatically reduce false positive detections.

More control experiments were performed to show the potential application of this nanopore assay in RNA detection and a complex biological environment. In Figure S8, the RNA target strand rA can also be recognized by the probes and result in a OF∼ 0.6 at the specific binding site, which indicates its potential for direct RNA detection without reverse transcription. Human total RNA was used to mimic the complex biological sample, providing a wide range of random RNA segments with various structures and lengths. The sensing platform kept working, as long as the blockade current caused by the random RNA was appropriately filtered before analysis of the events (Figure S9). Thus, the method offers a new means to analyze specific signals and target readout of nucleic acids in complex biological samples like total nucleic acid extracts.

## Conclusions

To conclude, a multiplexed nucleic acids detection method based on a split GQ and nanopore sensing technique was established. We found that four adjacent split GQs, formed upon the target strand binding, can be detected by the glass nanopore and separated from the background signal of G3 probes on the carrier. The split GQ reports the binding of the target strand by enhancing the nanopore signal. Benefiting from the split GQ sensing strategy, even a single base difference can be distinguished. Combining with the DNA carrier-based nanopore platform, multiplexed nucleic acid sensing is achieved without any crosstalk. Further applications for target strand sensing in a complex biological environment and RNA detection were also demonstrated.

Comparing with the split GQ based fluorescent, colorimetric, or electrochemical assays, the proposed nanopore method can detect multiple nucleic acid targets simultaneously without any modifications or ligands. Compared with other nanopore sensors, besides the feature of multiplexed sensing, the method using multiple GQ works with relatively large and hence easy to fabricate glass nanopores without any modification. In summary, this work supplies a multiplexed nucleic acid sensing method that may be useful for the tracking of viral infections. In the future, this programmable sensing platform could be developed into a screening system for several diseases in a single test.

## Supporting Information Available

Details of DNA sequences and supplementary data (PDF)
