# Supplementary material for: Split G-Quadruplexes Enhance Nanopore Signals for Simultaneous Identification of Multiple Nucleic Acids
Source: Nano Lett. 2022 Jun 7;22(12):4993–8. doi: 10.1021/acs.nanolett.2c01764 (PMC9228402; doi:10.1021/acs.nanolett.2c01764)
Supplement: Supplementary file 1 — nl2c01764_si_001.pdf [file nl2c01764_si_001.pdf]

Supporting Information for

Split G-quadruplexes enhance nanopore signals for simultaneous identification of multiple nucleic acids

Jinbo Zhu, Filip Bošković, Ulrich F. Keyser*

Cavendish Laboratory, University of Cambridge, JJ Thomson Avenue, Cambridge, CB3 0HE, United Kingdom

S1. Materials and experimental details

S1.1 Materials

DNA and RNA oligonucleotides were purchased from Integrated DNA Technologies, Inc. (IDT). The sequences of DNA strands are listed in Table S1-7. M13mp18 ssDNA, BamHI-HF and EcoRI-HF were purchased from New England Biolabs. Universal human reference RNA was provided by Thermo Fisher Scientific and NMM (N-methyl mesoporphyrin IX) was purchased from Sigma-Aldrich. Other chemicals were of reagent grade and were used without further purification.

S1.2 Fluorescence spectroscopy

To prepare the DNA sample for fluorescence measurement, 1 μM strand G3, G9 and S were heated at 88°C for 5 min in TMK buffer (10 mM Tris-HCl, 10 mM $MgCl_2$, 20 mM KCl, pH 8.0) and then slowly cooled down to room temperature (~20 °C). The resulting solution was mixed with 2 μM NMM. After incubation for 20 min, the fluorescence spectra were collected from 550 to 750 nm with the excitation wavelength of 399 nm. In order to investigate the effects of cations, TM (10 mM Tris-HCl, 10 mM $MgCl_2$, pH 8.0), TMK and TMLi (10 mM Tris-HCl, 10 mM $MgCl_2$, 100 mM LiCl, pH 8.0) buffer solutions were used to prepare the samples.

S1.3 Preparation of DNA carriers

The same 190 DNA oligonucleotides (staples) designed in our previous work[1] were used here. They need to be accurately mixed before preparation of the DNA carriers. For the DNA carriers with G3 probes in the middle (Figure 1, Figure 3 and Figure S2), staple strands 95-97 were replaced with the corresponding G3 probes (Table S2). When n = 3, staple 95-97 were replaced with MG1-3 and M4-6; when n = 4, staple 95-97 were replaced with MG1-4 and M5-6; when n = 5, staple 95-97 were replaced with MG1-5 and M6. To prepare the staples of the carrier for HBB mutation detection, staple 95-97 were replaced with MH1-4 and M5-6. For DNA carrier with DNA dumbbells and three kinds of G3 probes, staple 26-32 were replaced with dumbbell strands DA1-12 (Table S6); staple 82-88 were replaced with dumbbell strands DB1-12 (Table S7); the oligos to replace the staples at the three sensing sites (A, B and C) can be found in Table S10.

The carrier synthesis follows our previous work.[1] The 7228 nt DNA scaffold was linearized from M13mp18 ssDNA using the published protocol.[1] After mixing the staples corresponding to the design of relevant carrier, the cut M13 scaffold was added into the solution (20 nM M13 scaffold, 60 nM staples and 120 nM dumbbell strands or G3 probes) and heated to 70°C followed by a linear cooling ramp to 25°C over 50 minutes. Finally, the resulting solution was diluted with a washing buffer (10 mM Tris-HCl, 0.5 $MgCl_2$, pH 8.0) to 500 μL and centrifuged with an Amicon Ultra 100kDa filter to remove the excess DNA strands at 6000 g for 10 mins (repeated 3 times). About 35 μL solution of DNA carrier was obtained and quantified with NanoDrop 2000 spectrophotometer.

S1.4 Nanopore measurement

The fabrication and measurement of the glass nanopore follows the former study.[1] Glass nanopores with diameters 14±3 nm were generated on a laser-assisted pipette puller (P-2000, Sutter Instrument) by pulling quartz capillaries (outer diameter 0.5 mm and inner diameter 0.2 mm, Sutter Instrument). The resulting nanopores were fixed on a PDMS chip. 200 nM target strand and 240 nM G9 probes were premixed and then incubated with 2.5 nM DNA carrier in TMK buffer at room temperature for 10 minutes. Then carrier solution was diluted with Tris-LiCl buffer solution (10 mM Tris-HCl, 4 M LiCl, 20 mM KCl, pH 9.0) to 0.25 nM and then added to the tip side of the glass nanopore. Two electrodes were placed at the two sides of the nanopore. The electrodes were connected to an Axon Axopatch 200B amplifier (Molecular Devices), which applied a voltage of 600 mV to drive the DNA through nanopores and recorded the current signal. The current signal was filtered with an external Bessel filter (Frequency Devices) at 50 kHz and digitized at a 250 kHz sampling rate with a data card (PCI-6251, National Instruments). It normally took 1-2 hours for each measurement to get enough translocation events (>1000).

S1.5 Nanopore data analysis

Home-made LabVIEW algorithms were used for data collection and analysis. The raw data was analyzed by following the steps described in the previous works.[1,2] An example of the translocation event searching and cropping from the raw data is shown in Figure S10. About 30% of the total translocation events are unfolded. The barcode determination program[1] was applied to read the multiplexed sensing result, because the binding regions for barcodes were used here for the references and G3 probes on the carrier.

S1.6 Native polyacrylamide gel electrophoresis (PAGE)

Gel analysis was applied to verify the hybridization between target strand and G-rich probes. The DNA strands were annealed in TMK buffer before PAGE. 10 μL of 5 μM DNA was mixed with 6 × loading buffer, and then analyzed by 15 % (v/v) native polyacrylamide gel. The electrophoresis was conducted in 1 × TAE buffer (40 mM Tris, 20 mM acetic acid, 1 mM EDTA, pH 8.0) with 20 mM KCl at a constant voltage of 110V (10 V/cm) for about 1 hour and stained by GelRed.

S2. Figures

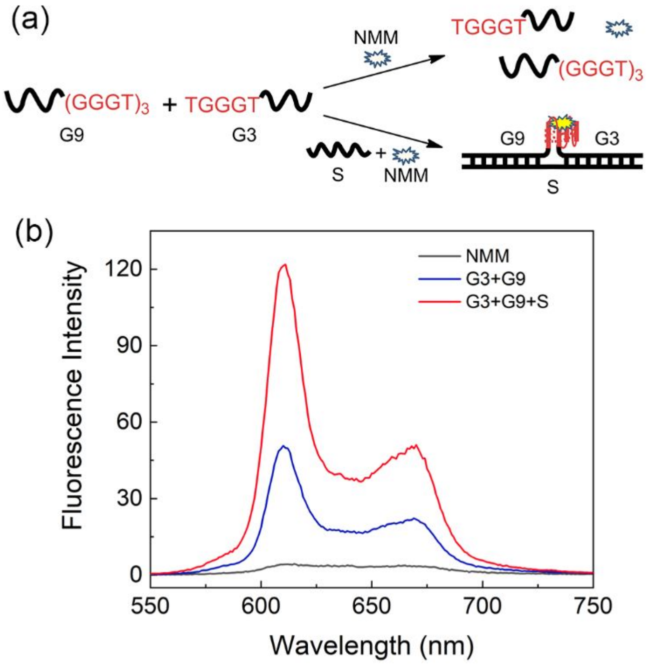

Figure S1. Detection of split GQ formed upon target strand S hybridization by fluorescent dye NMM (N-methyl mesoporphyrin IX). (a) Scheme of the split GQ-based fluorescent sensing method. NMM is a GQ specific binding dye, whose fluorescence intensity can be dramatically enhanced by GQ binding. (b) Fluorescence spectra of NMM (2 μM) with S·G3·G9 DNA complex (1 μM) or mixture of G3 and G9 probes (1 μM) in TMK buffer solutions (10 mM Tris, 10 mM $MgCl_2$, 20 mM KCl, pH 8.0) were recorded by exciting the samples at 399 nm.

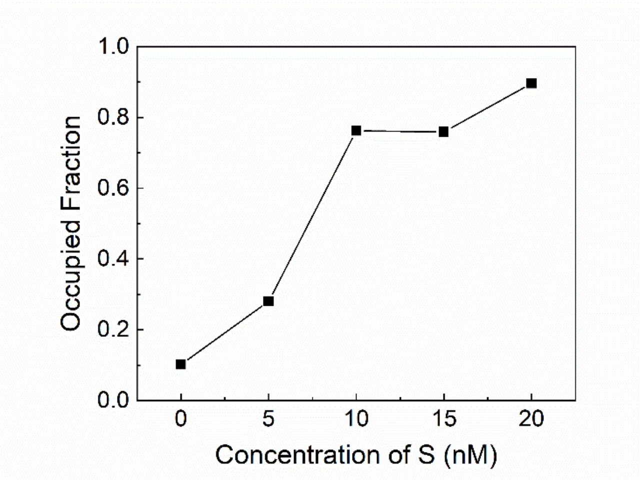

Figure S2. Effect of the concentration of target S on the occupied factions at the center binding site of the carrier with four G3 probes. The concentration of the carrier was kept at 0.25 nM for all nanopore measurements. Statistics of the translocation events can be found in Table S8.

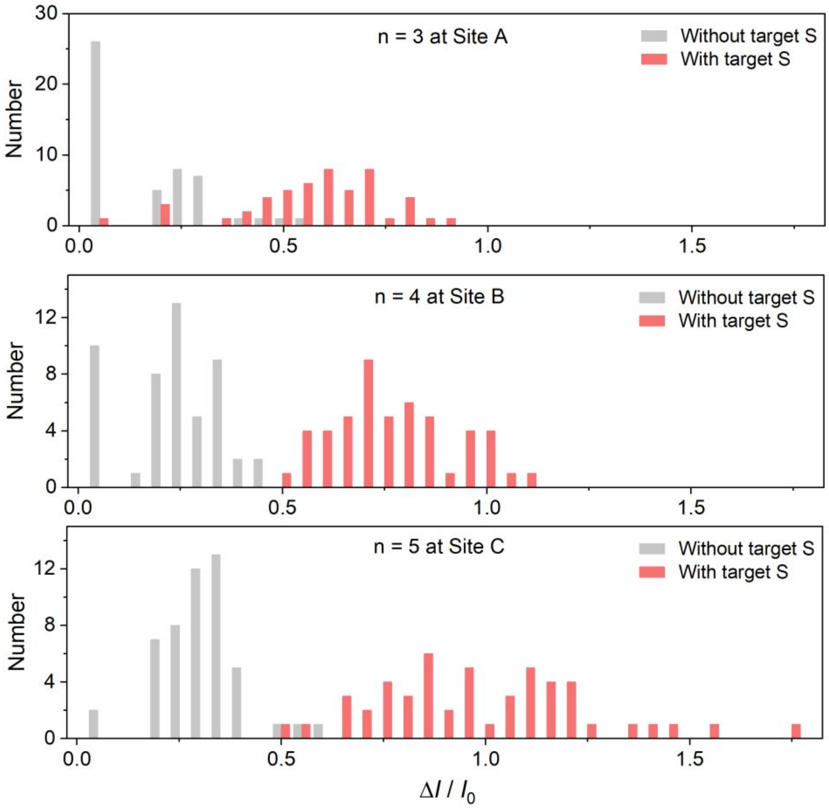

Figure S3. Nanopore relative peak intensities ($\Delta I/I_0$) distribution histogram of DNA structures at sensing site A (top), B (middle), and C (bottom) of DNA carrier in Figure 2 with (red) or without (grey) target S. 50 unfolded events for both samples are used for analysis. The concentrations of DNA carrier, strand S and G9 probe were 0.25 nM, 60 nM and 72 nM, respectively.

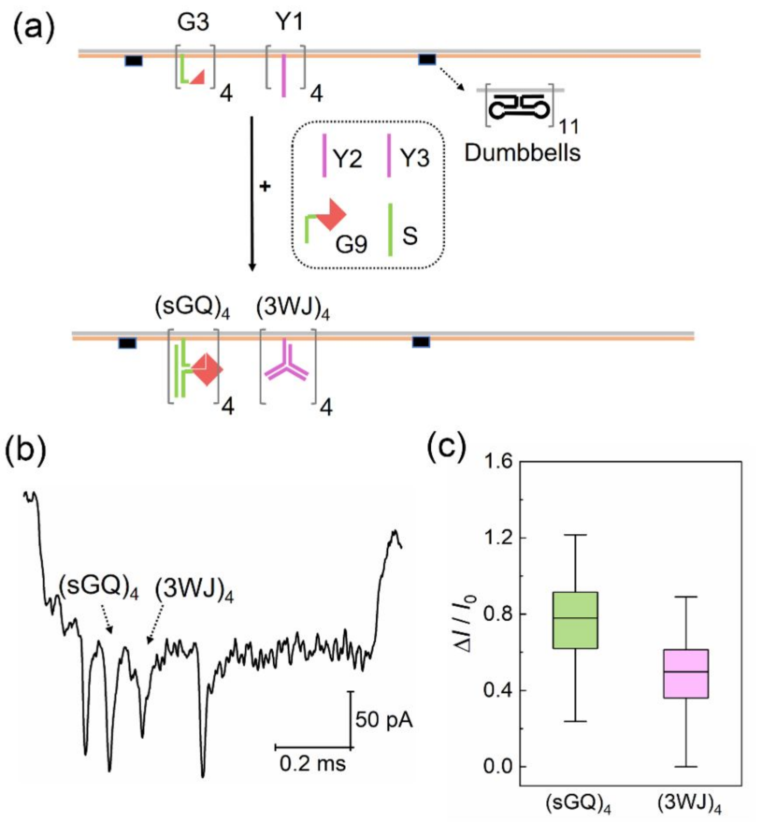

Figure S4. Comparison of split GQ and DNA three-way junction on a same DNA carrier. (a) Design of the carrier for DNA structure comparison. Four adjacent G3 probes and four Y1 overhangs (Table S4) were placed at two sensing sites on the carrier. After the addition of other components (in the dashed box), two groups of $(sGQ)_4$ and $(3WJ)_4$ in the similar molecular weights were formed on the carrier. (b) Typical nanopore current signals of the carrier with two kinds of DNA structures. (c) Box chart showing the relative peak intensities ($\Delta I/I_0$) of $(sGQ)_4$ and $(3WJ)_4$ at the two sensing sites based on the analysis of 86 unfolded events. I_0 = 0.130 nA. The mean values are represented by a line across the box. 0.25 nM carrier was mixed with 60 nM S, 72 nM G9, 60 nM Y2 and 60 nM Y3.

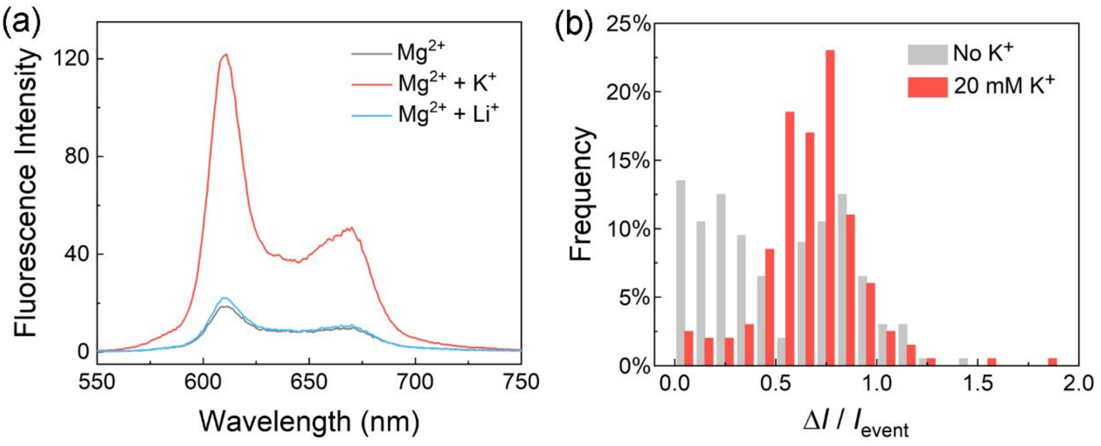

Figure S5. Effect of potassium ion on the detection of split GQ by (a) fluorescent dye NMM and (b) solid state nanopore. (a) Fluorescence spectra of NMM (2 μM) and S·G3·G9 DNA complex (1 μM) in Tris buffer solutions (10 mM Tris, 10 mM $MgCl_2$, pH 8.0) with different cations (20 mM

KCl or 100 mM LiCl) were recorded by exciting the samples at 399 nm. (b) Distributions of the second current drop ΔI of 206 unfolded events with 20 mM K^+ (red bars) and 200 unfolded events without K^+ (gray bars). The data are from the carrier with four split GQs in the middle.

Hbbm	+	−	−	+	+	+
G9h	−	+	−	−	+	+
MH1	−	−	+	+	−	+

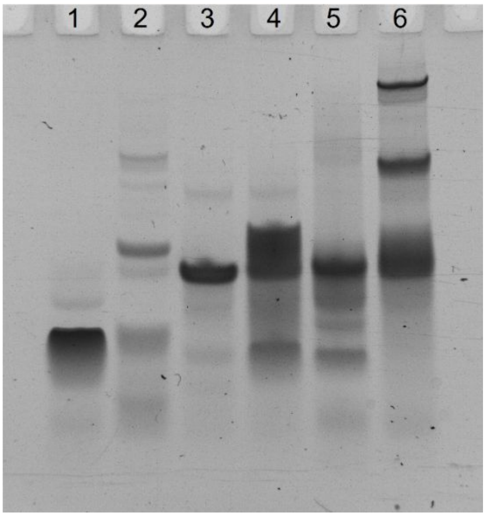

Figure S6. Hybridization between target strand Hbbm and G-rich probes, G9h and MH1 (G3 probe on carrier), analyzed by 15 % (v/v) native PAGE in 1 × TAE buffer with 20 mM KCl. The content of the mixture loaded into each lane is indicated in the table above the gel. The concentration is 5 μM for all DNA strands. Intermolecular self-assembly of G9h in K^+ contained solution[3,4] causes the appearance of slower bands in lane 2 compared to lane 1 in spite of the similar lengths of Hbbm and G9h. In lane 6, disappearance of the band of Hbbm and appearance of new bands at the top compared to lane 4 and 5 prove the hybridization between Hbbm and G-rich probes. Stacking of the GQ may happen at this concentration and result in more than one band at the top in lane 6.[5]

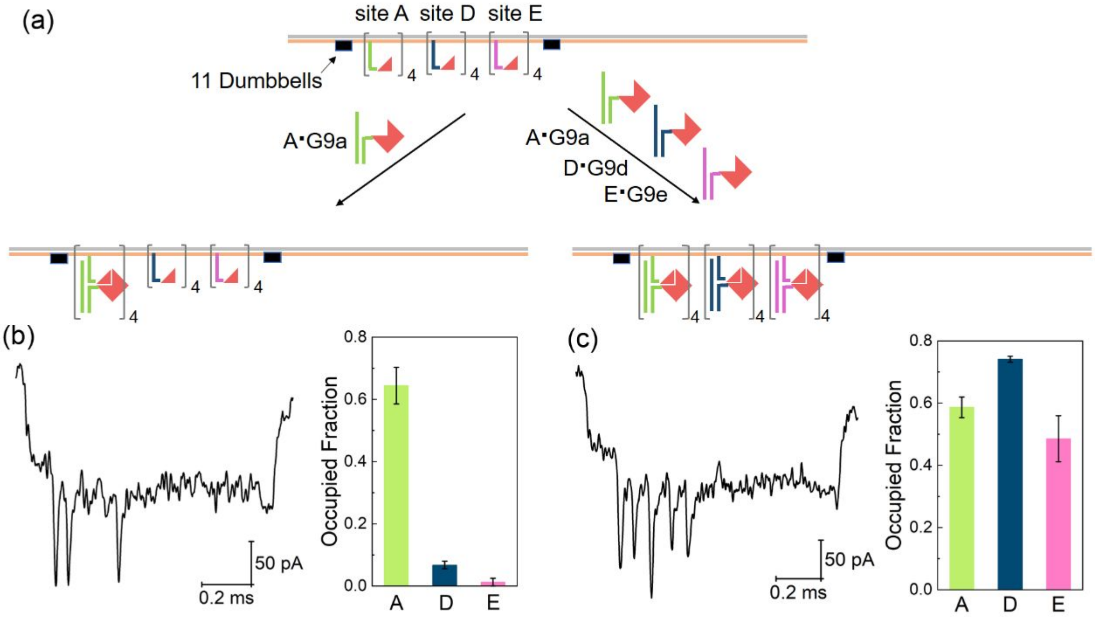

Figure S7. Multiplex detection of three DNA target strands A, D and E using one carrier with three groups of G3 probes. Construct of the carrier is shown in (a). G3 probes on the three binding sites A, D and E on the carrier and G9 probes G9a, G9d, and G9e in the solution are designed to hybridize with the target strands A, D and E in the sample, respectively. Results and example event for the detection of strand A are given in (b), and the results for the detection of three targets are given in (c). The concentrations of DNA carrier and target strands in the nanopore measurement are 0.25 nM and 20 nM, respectively. The standard error bars are obtained from three independent measurements. Statistics of the translocation events can be found in Table S9.

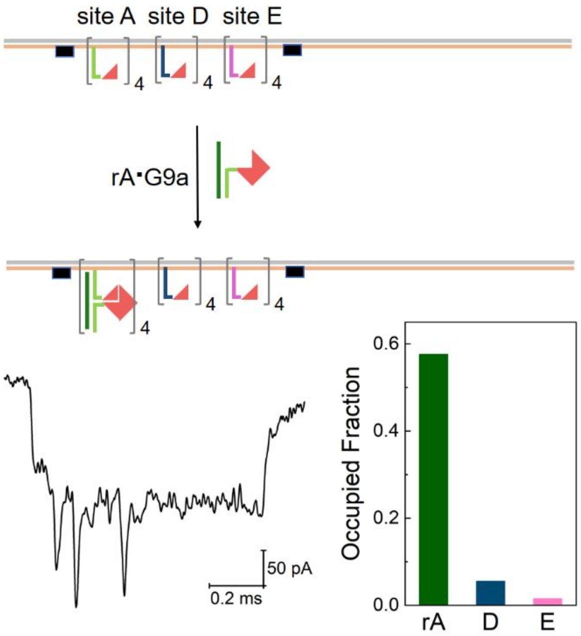

Figure S8. Detection of RNA strand rA by the split GQ based nanopore sensor. The same carrier in Figure 4 is used here. Nanopore measurement was performed in the Tris-LiCl buffer solution (10 mM Tris-HCl, 4 M LiCl, 20 mM KCl, pH 9.0). The concentrations of DNA carrier, strand rA and G9a were 0.25 nM, 20 nM and 24 nM respectively in the nanopore measurement. Statistics of the translocation events can be found in Table S9.

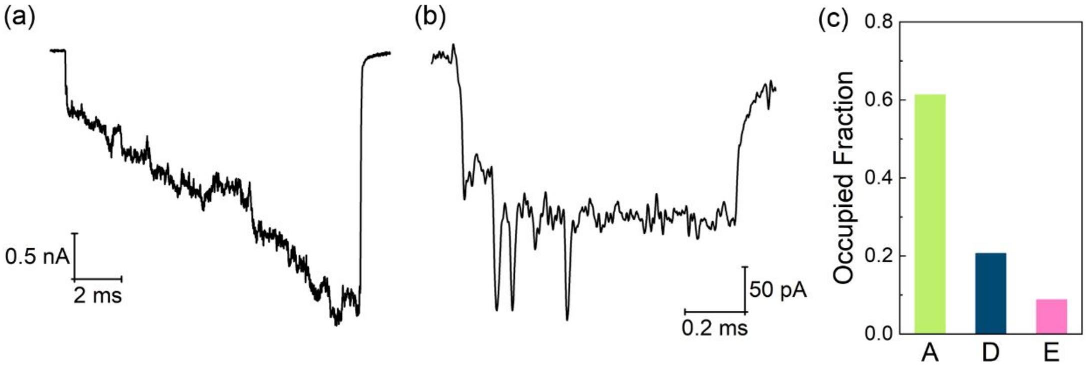

Figure S9. Detection of strand A by the split GQ based nanopore sensor in complex biological environment with human total RNA. (a) Sample noise signal caused by RNA background. (b)

Sample translocation event of unfolded DNA carrier. (c) Bar graph of the occupied fractions for different binding sites. Statistics of the translocation events can be found in Table S9. The concentrations of carrier, strand A and reference RNA are 0.168 nM, 6.67 nM and 66.7 ng/μL, respectively.

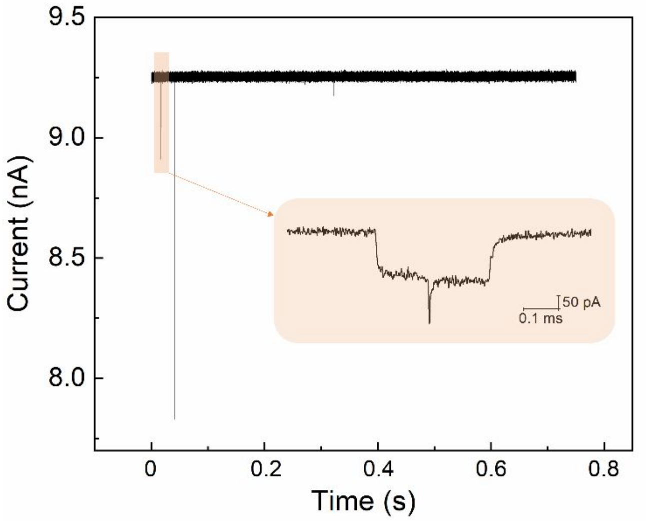

Figure S10. Example of the translocation event searching and cropping from the nanopore raw data collected from DNA carrier in Figure 1 with target S. The translocation event was cropped and enlarged in the orange box.

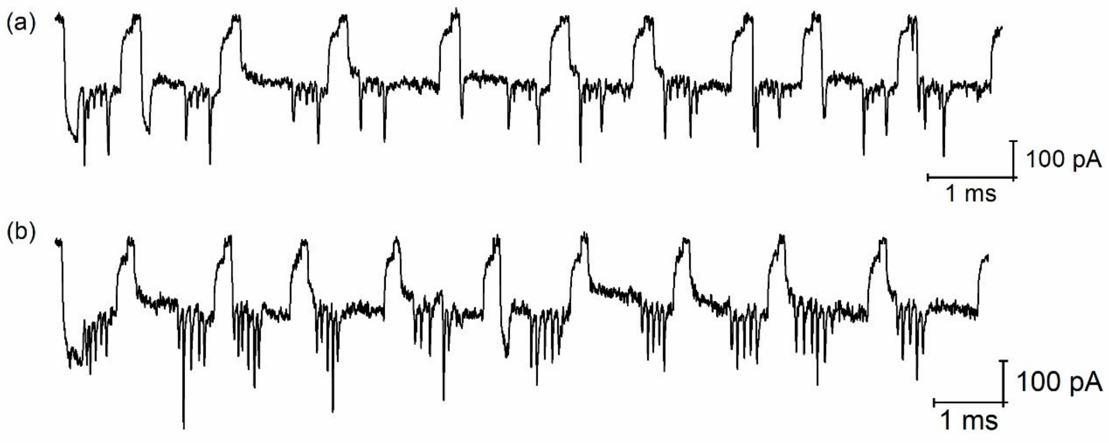

Figure S11. First ten translocation events of DNA carrier in Figure 2 (a) without target S and (b) with target S.

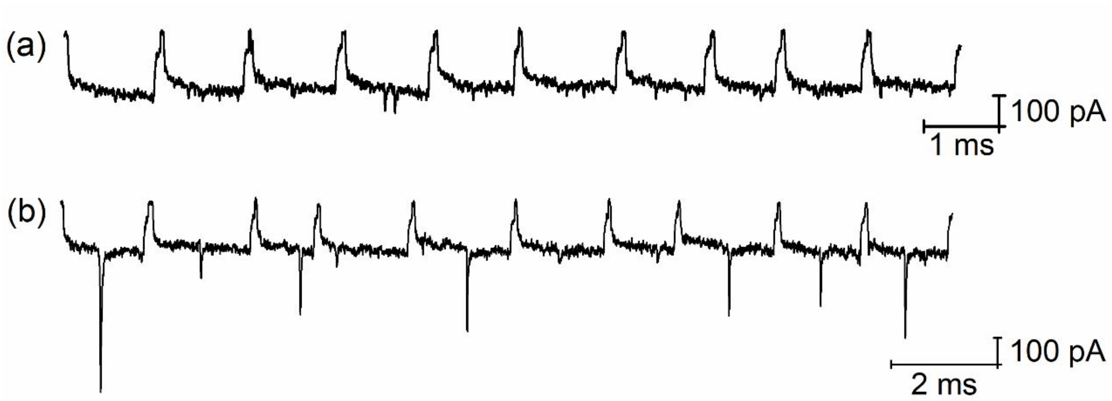

Figure S12. First ten translocation events of DNA carrier in Figure 3 (a) with HBBw and (b) with HBBm.

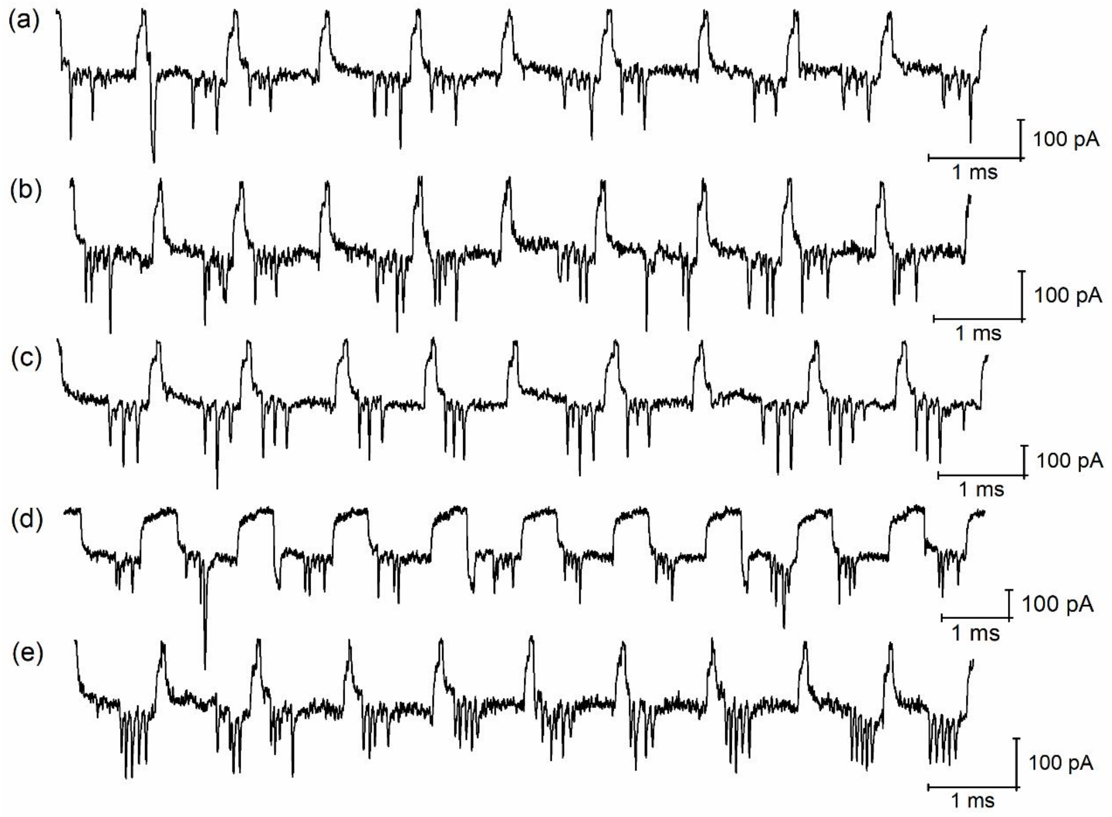

Figure S13. First ten translocation events of DNA carrier in Figure 4 (a) without any target, (b) only with target A, (c) only with target B, (d) only with target C, and (e) with all three targets.

S3. Tables

Table S1. Sequences of target nucleic acid strands and G segments.

Strand	Sequence
S	TGATTGTGAA GAAGAAGAGT
G3	ACTCTTCTTC TGGGT
G9	GGGTGGGTGGGT TTCACAATCA
HBBw	GTGCACCTGACTC CTGAGGAGAAG
HBBm	GTGCACCTGACTC CTGTGGAGAAG
G9h	GGGTGGGTGGGT GAGTCAGGTGCAC
A[a]	TGATTGTGAA GAAGAAGAGT
B	CGACTACACTCT CGATGAAGAA
C	CATACTGAGAAC TCAAGAGTCT
D	AAGAAAGGAG CTAAATTGTT
E	TGGTGTTTAT TCTGTTATTT
rA	r(UGAUUGUGAA UAAGAAGAGU)
G9a	GGGTGGGTGGGT TTCACAATCA
G9b	GGGTGGGTGGGT AGAGTGTAGTCG
G9c	GGGTGGGTGGGT GTTCTCAGTATG
G9d	GGGTGGGTGGGT CTCCTTTCTT
G9e	GGGTGGGTGGGT ATAAACACCA
Y2	GTCTCATTCT AGTACATGTG
Y3	CACATGTACT CACATCCTCA

[a] To simplify the design, we use the sequence of strand S as target A.

Table S2. Strands to replace oligos 95, 96 and 97 on carrier. MGx and MHx are G3 probes for target S and HBBm, respectively.

Strand	Sequence
MG1	GAAATTATTCATTAAAGGTG TT ACTCTTCTTC TGGGT
MG2	AATTATCACCGTCACCGACT TT ACTCTTCTTC TGGGT
MG3	TGAGCCATTTGGGAATTAGA TT ACTCTTCTTC TGGGT
MG4	GCCAGCAAAATCACCAGTAG TT ACTCTTCTTC TGGGT
MG5	CACCATTACCATTAGCAAGG TT ACTCTTCTTC TGGGT
MH1	GAAATTATTCATTAAAGGTG TT CTTCTCCACAG TGGGT
MH2	AATTATCACCGTCACCGACT TT CTTCTCCACAG TGGGT
MH3	TGAGCCATTTGGGAATTAGA TT CTTCTCCACAG TGGGT
MH4	GCCAGCAAAATCACCAGTAG TT CTTCTCCACAG TGGGT
M4	GCCAGCAAAATCACCAGTAG
M5	CACCATTACCATTAGCAAGG
M6	CCGGAAACGTCACC

Table S3. Strands to replace oligos 42, 43 and 44 at sensing site A on carrier. AGx are G3 probes

for target A.

Strand	Sequence
AG1	TTCGACAACTCGTATTAAAT TT ACTCTTCTTC TGGGT
AG2	CCTTTGCCCGAACGTTATTA TT ACTCTTCTTC TGGGT
AG3	ATTTTAAAAGTTTGAGTAAC TT ACTCTTCTTC TGGGT
AG4	ATTATCATTTTGCGGAACAA TT ACTCTTCTTC TGGGT
A4	ATTATCATTTTGCGGAACAA
A5	AGAAACCACCAGAAGGAGCG
A6	GAATTATCATCATA

Table S4. Strands to replace oligos 56, 57 and 58 at sensing stie B on carrier. BGx, BAGx and DGx are G3 probes for target B, A and D, respectively.

Strand	Sequence
BG1	AATTAATTTTCCCTTAGAAT TT TTCTTCATCG TGGGT
BG2	CCTTGAAAACATAGCGATAG TT TTCTTCATCG TGGGT
BG3	CTTAGATTAAGACGCTGAGA TT TTCTTCATCG TGGGT
BG4	AGAGTCAATAGTGAATTTAT TT TTCTTCATCG TGGGT
BAG1	AATTAATTTTCCCTTAGAAT TT ACTCTTCTTC TGGGT
BAG2	CCTTGAAAACATAGCGATAG TT ACTCTTCTTC TGGGT
BAG3	CTTAGATTAAGACGCTGAGA TT ACTCTTCTTC TGGGT
BAG4	AGAGTCAATAGTGAATTTAT TT ACTCTTCTTC TGGGT
DG1	AATTAATTTTCCCTTAGAAT TT AACAATTTAG TGGGT
DG2	CCTTGAAAACATAGCGATAG TT AACAATTTAG TGGGT
DG3	CTTAGATTAAGACGCTGAGA TT AACAATTTAG TGGGT
DG4	AGAGTCAATAGTGAATTTAT TT AACAATTTAG TGGGT
BY1	AATTAATTTTCCCTTAGAAT TGAGGATGTG AGAATGAGAC
BY2	CCTTGAAAACATAGCGATAG TGAGGATGTG AGAATGAGAC
BY3	CTTAGATTAAGACGCTGAGA TGAGGATGTG AGAATGAGAC
BY4	AGAGTCAATAGTGAATTTAT TGAGGATGTG AGAATGAGAC
B5	CAAAATCATAGGTCTGAGAG
B6	ACTACCTTTTTAAC

Table S5. Strands to replace oligos 70, 71 and 72 at sensing site C on carrier. CGx, CAGx and EGx are G3 probes for target C, A and E, respectively.

Strand	Sequence
CG1	GAACGCGCCTGTTTATCAAC TT AGACTCTTGA TGGGT
CG2	AATAGATAAGTCCTGAACAA TT AGACTCTTGA TGGGT
CG3	GAAAAATAATATCCCATCCT TT AGACTCTTGA TGGGT
CG4	AATTTACGAGCATGTAGAAA TT AGACTCTTGA TGGGT
CAG1	GAACGCGCCTGTTTATCAAC TT ACTCTTCTTC TGGGT
CAG2	AATAGATAAGTCCTGAACAA TT ACTCTTCTTC TGGGT
CAG3	GAAAAATAATATCCCATCCT TT ACTCTTCTTC TGGGT

CAG4	AATTTACGAGCATGTAGAAA TT ACTCTTCTTC TGGGT
CAG5	CCAATCAATAATCGGCTGTC TT ACTCTTCTTC TGGGT
EG1	GAACGCGCCTGTTTATCAAC TT AAATAACAGA TGGGT
EG2	AATAGATAAGTCCTGAACAA TT AAATAACAGA TGGGT
EG3	GAAAAATAATATCCCATCCT TT AAATAACAGA TGGGT
EG4	AATTTACGAGCATGTAGAAA TT AAATAACAGA TGGGT
C5	CCAATCAATAATCGGCTGTC
C6	TTTCCTTATCATTC

Table S6. Strands of dumbbells to replace oligos 26-32 on carrier.

Strand	Sequence
DA1	CTGAAAGCGTAAGAATACGTGGCACAGACAATATTTTTGAATGGCT
DA2	ACATCACTTGTCCTCTTTTGAGGAACAAGTTTTCTTGTCCTGAGTAGA
DA3	AGAACTCAAATCCTCTTTTGAGGAACAAGTTTTCTTGTCTATCGGCCT
DA4	TGCTGGTAATTCCTCTTTTGAGGAACAAGTTTTCTTGTATCCAGAACA
DA5	ATATTACCGCTCCTCTTTTGAGGAACAAGTTTTCTTGTCAGCCATTGC
DA6	AACAGGAAAATCCTCTTTTGAGGAACAAGTTTTCTTGTACGCTCATGG
DA7	AAATACCTACTCCTCTTTTGAGGAACAAGTTTTCTTGTATTTTGACGC
DA8	TCAATCGTCTTCCTCTTTTGAGGAACAAGTTTTCTTGTGAAATGGATT
DA9	ATTTACATTGTCCTCTTTTGAGGAACAAGTTTTCTTGTGCAGATTCAC
DA10	CAGTCACACGTCCTCTTTTGAGGAACAAGTTTTCTTGTACCAGTAATA
DA11	AAAGGGACATTCCTCTTTTGAGGAACAAGTTTTCTTGTTCTGGCCAAC
DA12	AGAGATAGAATCCTCTTTTGAGGAACAAGTTTTCTTGTCCCTTCTGAC

Table S7. Strands of dumbbells to replace oligos 82-88 on carrier.

Strand	Sequence
DB1	AGATAGCCGAACAAAGTTACCAGAAGGAAACCGAGGAAACGCAATA
DB2	AAAAATGAAATCCTCTTTTGAGGAACAAGTTTTCTTGTATAGCAGCCT
DB3	TTACAGAGAGTCCTCTTTTGAGGAACAAGTTTTCTTGTAATAACATAA
DB4	AAACAGGGAATCCTCTTTTGAGGAACAAGTTTTCTTGTGCGCATTAGA
DB5	CGGGAGAATTTCCTCTTTTGAGGAACAAGTTTTCTTGTAACTGAACAC
DB6	CCTGAACAAATCCTCTTTTGAGGAACAAGTTTTCTTGTGTCAGAGGGT
DB7	AATTGAGCGCTCCTCTTTTGAGGAACAAGTTTTCTTGTTAATATCAGA
DB8	GAGATAACCCTCCTCTTTTGAGGAACAAGTTTTCTTGTACAAGAATTG
DB9	AGTTAAGCCCTCCTCTTTTGAGGAACAAGTTTTCTTGTAATAATAAGA
DB10	GCAAGAAACATCCTCTTTTGAGGAACAAGTTTTCTTGTATGAAATAGC
DB11	AATAGCTATCTCCTCTTTTGAGGAACAAGTTTTCTTGTTTACCGAAGC
DB12	CCTTTTTAAGTCCTCTTTTGAGGAACAAGTTTTCTTGTAAAAGTAAGC

Table S8. Statistics of translocation events of carriers with G3 probes in the middle.

Sample	I_0	Total number of	Number of events	Occupied fraction

		unfolded events	with peak[a]	
4xG3+G9 (Figure 1)	0.119 nA	103	16	15.5%
4xG3+S+G9 (Figure 1)	0.129 nA	405	363	89.6%
4xG3h+HBBw+G9h_repeat 1 (Figure 3)	0.118 nA	141	16	11.3%
4xG3h+HBBw+G9h_repeat 2 (Figure 3)	0.157 nA	268	35	13.1%
4xG3h+HBBw+G9h_repeat 3 (Figure 3)	0.179 nA	280	31	11.1%
4xG3h+HBBm+G9h_repeat 1 (Figure 3)	0.137 nA	271	191	70.5%
4xG3h+HBBm+G9h_repeat 2 (Figure 3)	0.161 nA	242	202	83.5%
4xG3h+HBBm+G9h_repeat 3 (Figure 3)	0.164 nA	234	140	59.8%
4xG3 (Figure S2)	0.124 nA	98	10	10.2%
4xG3+5 nM A·G9a (Figure S2)	0.132 nA	193	54	28.0%
4xG3+10 nM A·G9a (Figure S2)	0.132 nA	541	412	76.2%
4xG3+15 nM A·G9a (Figure S2)	0.152 nA	212	161	75.9%
4xG3+20 nM A·G9a (Figure S2)	0.129 nA	405	363	89.6%

[a] Peaks are judged by threshold 0.3 ($\Delta I/I_0$).

Table S9. Statistics of translocation events of carriers with three kinds of G3 probes between two groups of dumbbells on the carrier.

Sample name	I_0	Total number of unfolded events	Number of peaks at site A	Number of peaks at site B or D	Number of peaks at site C or E
4xG3abc+G9a+G9b+G9c (Blank, 3 repeats, Figure 4)	0.117 nA	157	9	13	4
	0.110 nA	94	3	4	1
	0.156 nA	167	10	28	9
4xG3abc+A+G9a+G9b+G9c (Only A, 3 repeats, Figure 4)	0.122 nA	424	302	23	10
	0.136 nA	172	147	13	5
	0.135 nA	926	713	90	43
4xG3abc+B+G9a+G9b+G9c (Only B, 3 repeats, Figure 4)	0.128 nA	180	41	114	3
	0.183 nA	113	4	79	8
	0.135 nA	239	13	146	25
4xG3abc+C+G9a+G9b+G9c (Only C, 3 repeats, Figure 4)	0.148 nA	156	5	18	115
	0.123 nA	126	10	4	91
	0.121 nA	103	4	6	53
4xG3abc+A+B+C+G9a+G9b +G9c (ABC, 3 repeats, Figure 4)	0.168 nA	561	497	474	431
	0.126 nA	130	110	86	89
	0.125 nA	487	394	353	356
4xG3ade+A+G9a	0.148 nA	407	233	34	15

(3 repeats, Figure S6)	0.192 nA	45	27	2	0
	0.128 nA	67	51	5	0
4xG3ade+A+D+E+G9a+G9d +G9e (3 repeats, Figure S6)	0.145 nA	193	124	145	122
	0.140 nA	56	33	42	22
	0.139 nA	72	38	52	31
4xG3ade+rA+G9a (Figure S7)	0.149 nA	198	114	11	3
4xG3ade+A+G9a with human reference RNA (Figure S8)	0.157 nA	194	119	40	17

Table S10. Strands to replace the staple oligos at the three sensing sites (A, B and C) for preparing different DNA carriers.

	Site A (staple 42-44)	Site B (staple 56-58)	Site C (staple 70-72)
Carrier in Figure 2	AG1-3, A4-6	BAG1-4, B5-6	CAG1-5, C6
Carrier in Figure 4	AG1-4, A5-6	BG1-4, B5-6	CG1-4, C5-6
Carrier in Figure S7	AG1-4, A5-6	DG1-4, B5-6	EG1-4, C5-6
Carrier in Figure S4	AG1-4, A5-6	BY1-4, B5-6	none

Reference

[1] Bell, N. A. W.; Keyser, U. F. Digitally encoded DNA nanostructures for multiplexed, single-molecule protein sensing with nanopores. *Nat. Nanotechnol.* **2016,** *11*, 645.

[2] Zhu, J.; Ermann, N.; Chen, K.; Keyser, U. F. Image encoding using multi-level DNA barcodes with nanopore readout. *Small* **2021,** *17* (28), 2100711.

[3] Zhu, J.; Zhang, L.; Dong, S.; Wang, E., How to split a G-quadruplex for DNA detection: new insight into the formation of DNA split G-quadruplex. *Chemical Science* **2015,** *6* (8), 4822-4827.

[4] Lv, M.; Guo, Y.; Ren, J.; Wang, E. Exploration of intramolecular split G-quadruplex and its analytical applications. *Nucleic Acids Res.* **2019,** *47* (18), 9502-9510.

[5] Do, N. Q.; Lim, K. W.; Teo, M. H.; Heddi, B.; Phan, A. T. Stacking of G-quadruplexes: NMR structure of a G-rich oligonucleotide with potential anti-HIV and anticancer activity. *Nucleic Acids Res.* **2011,** *39* (21), 9448-9457.
